# Supplementary material for: Molecular Docking Guided Grid-Independent Descriptor Analysis to Probe the Impact of Water Molecules on Conformational Changes of hERG Inhibitors in Drug Trapping Phenomenon
Source: Int J Mol Sci. 2019 Jul 10;20(14):3385. doi: 10.3390/ijms20143385 (PMC6678931; doi:10.3390/ijms20143385)
Supplement: Supplementary file 1 [file ijms-20-03385-s001.zip › ijms-523333 sp for proof/suppl.pdf]

Supplementary Information

Probing the impact of water molecules on conformational changes of hERG inhibitors in drug trapping phenomenon

Saba Munawar[1] , Jamie I. Vandenberg[2] and Ishrat Jabeen[1*]

[1] Research Center for Modeling and Simulation (RCMS), National University of Science and Technology, Sector H-12, 44000, Islamabad Pakistan
[2] Victor Chang Cardiac Research Institute, Lowy Packer Building, 405 Liverpool Street Darlinghurst Sydney NSW 2010, Australia
* Correspondence: **Dr. Ishrat Jabeen** Associate Professor. *E-mail: ishrat.jabeen@rcms.nust.edu.pk Phone: +92-51-90855732. Research Center for Modeling and Simulation (RCMS). National University of Sciences and Technology (NUST). Sector H-12, Islamabad, Pakistan

Table of Content

**Table**	**Title**
Figure S1	The plot of energy minimization, time steps versus energy values.
Figure S2	RMSD values of hERG cryo-EM open conformational structure.
Table S1	GRIND models validation values of internal testset.
Table S2	GRIND models validation values of external testset.

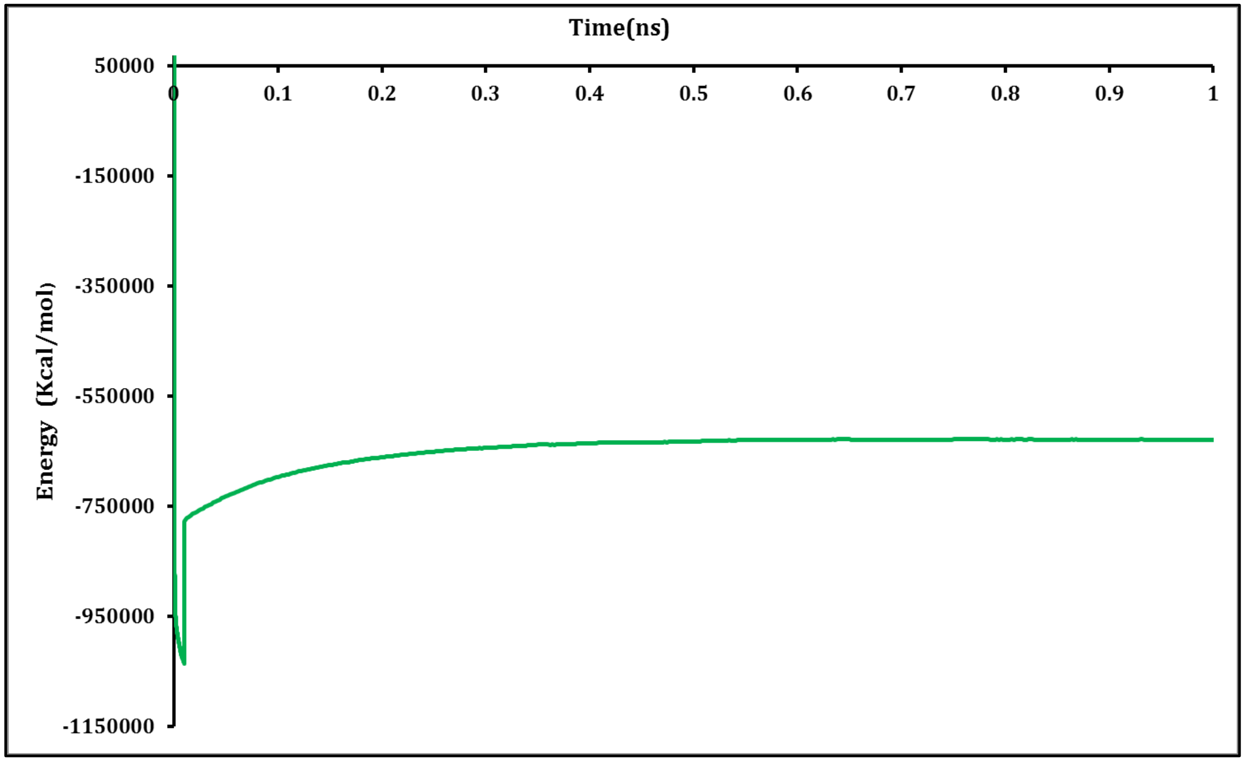

Figure S1: Plot showing energy minimization time steps versus energy values.

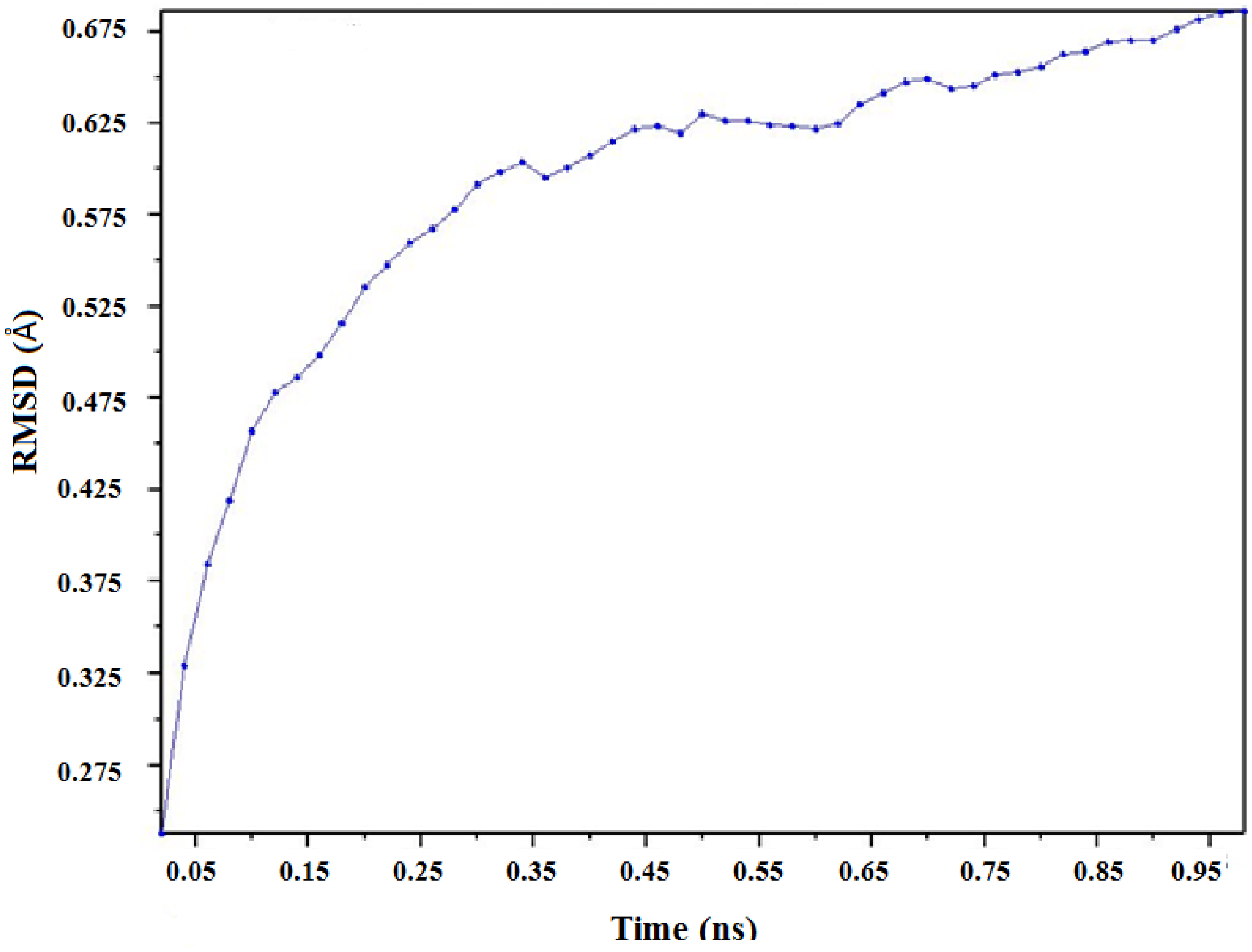

Figure S2: Plot showing RMSD values of hERG cryo-EM open conformational structure from starting frame to last frame

Table S1: Showing actual and predicted values of internal test set compounds obtained from open and closed solvated and non-solvated conformations.

Compounds	Actual pIC_{50}	Solvated-Open	Non-Solvated-Open	Non-Solvated-Closed	Solvated-Closed
Desloratin	5.35	6.15	6.14	5.86	6.22
Carbamazepine	3.60	5.49	5.17	5.12	5.45
Phenytoin	3.62	4.33	3.34	4.53	4.77
Ropinirole	5.92	5.59	6.04	5.37	4.97
Cibenzoline	5.43	5.48	5.17	5.39	5.64
Protriptyline	5.93	6.12	6.17	5.18	5.55
Pilsicainide	4.69	5.52	4.97	5.39	5.52
BMCL_03_13_1829-1835_22	5.46	5.79	5.92	5.78	6.32
Doxepin	5.19	5.80	5.48	5.29	6.19
Imipramine	5.47	6.29	6.33	5.50	5.92
BMCL_03_13_1829-1835_16	4.59	6.02	6.05	5.84	6.45
Ondasetron	6.09	5.44	5.42	5.91	6.01
N-desmethyl-olanzapine	4.85	5.31	5.65	5.13	4.38
Metoclopramide	5.27	5.63	4.53	5.30	5.65
BMCL_03_13_1829-1835_21_1	5.66	6.06	5.92	5.54	5.61
Methadone(R)	5.15	5.63	5.28	5.10	5.56
Granisetron	5.43	4.46	5.65	5.02	5.21
N-desmethyl-clozapine	5.35	6.00	5.94	5.48	5.62
BMCL_03_13_1829-1835_17	5.83	6.08	6.11	5.90	6.41
Chlorpromazine	5.81	5.98	6.21	5.54	6.01
Cyamemazine	6.33	6.77	6.84	6.17	6.15
Citalopram	5.40	6.89	6.92	6.31	5.69
Norfluoxetine	5.60	6.18	5.76	5.62	5.83
Ajmaline	5.98	5.12	5.08	4.84	4.68
Clozapine	5.60	5.81	5.10	5.76	5.55

2-Hydroxymethyl-olanzapine	4.94	5.06	5.45	5.02	4.95
Ambasilide	5.44	6.33	6.02	6.05	6.09
Fentanyl	5.74	6.82	6.69	6.60	6.93
Trazodone	5.54	6.40	5.91	6.58	6.70
Clebopride	6.21	7.29	6.73	7.08	6.02
CJ-033466	5.59	7.05	7.11	5.96	5.11
clomiphene	6.74	7.52	7.05	7.05	6.77
BMCL_03_13_1829-1835_3	8.00	7.99	7.51	7.71	8.48
Trifluoperazine	5.85	6.32	5.97	5.68	6.16
JMC20081730_1	6.02	6.40	6.35	6.35	5.91
Risperidone	6.82	5.88	6.09	6.06	6.17
JMC20081730_23	5.73	7.10	7.44	6.41	6.14
Sertindole	8.00	8.12	8.13	7.85	7.83
Dofetilide	8.00	7.20	7.81	5.21	5.14
Gbr-12909/ vanoxerine	9.00	7.54	7.66	7.78	7.65
**R^2**		**0.58**	**0.52**	**0.48**	**0.36**

Table S2: Showing actual and predicted values of internal test set compounds obtained from open and closed solvated and non-solvated conformations

Sr. No	Compound ID	Smiles	Expimental pIC$_{50}$	hERG cryo_EM model in open state		hERG homology model in the closed state		Ref
				Non-Solvated	Solvated	Non-Solvated	Solvated	
1	CHEMBL3885379	FC(F)(F)c1c(OC[C@H]2C[C@@H]2c3ccc(Cl)cc3)ccn4c(CC5	6.42	7.09	7.51	6.37	6.33	[1]

		CC5)nnc14						
2	CHEMBL3897030	Brc1ccc2c(NC3=NC[C@@]4(CN5CC4CC5)O3)ncnn12	5.60	5.91	5.83	5.98	5.32	[2]
3	CHEMBL3899686	COc1ccc(OC)c(Cc2nc3ccccc3n2CC(=O)Nc4cc(cc(c4)C(C)(C)C)C(C)(C)C)c1	7.70	6.80	7.89	7.62	6.88	[3]
4	CHEMBL3904792	Cl.NC(=N)N\N=C\c1ccc(cc1)c2ccc(\C=N\Nc3ccc(F)cc3)cc2	5.48	6.52	6.59	6.99	6.58	[4]
5	CHEMBL3913789	Cl.NC(=N)N\N=C\c1ccc(cc1)c2ccc(\C=N\Nc3ccccc3Cl)cc2	5.94	7.26	6.94	6.42	6.54	[4]
6	CHEMBL3922790	CCN1CCCC1CNC(=O)c2ccc(Cn3c(Cc4c(Cl)cccc4Cl)nc5ccccc35)cc2	5.80	5.43	7.74	6.15	7.00	[3]
7	CHEMBL3923854	Cl.NC(=N)N\N=C\c1ccc(cc1)c2ccc(\C=N\Nc3ccc(F)cc3F)cc2	5.65	6.30	6.93	5.75	5.91	[4]
8	CHEMBL3941777	Cl.NC(=N)N\N=C\c1ccc(cc1)c2ccc(\C=N\Nc3ccccc3Br)cc2	6.36	7.02	8.04	7.25	7.18	[4]
9	CHEMBL3944559	Cl.Cl.C\C(=N/NC(=N)N)\c1ccc2c(Cc3cc(ccc23)\C(=N\NC(=N)N)\C)c1	5.95	6.36	7.10	7.41	6.91	[4]
10	CHEMBL3947919	CN(C)CCCNC(=O)c1ccc(Cn2c(Cc3c(Cl)cccc3Cl)nc4ccccc24)cc1	5.34	6.32	7.26	7.55	6.71	[3]

11	CHEMBL5931690	S1C=C([NH+](c2ccccc2)[C-]1\C=C(\Nc1ccc(OC)cc1)/C)c1ccccc1	7.14	8.14	8.13	7.96	7.43	[5]
12	Compound 3	C1=CC=C(C(=C1)C2=NC3=CN(C=CC3=N2)CC4=NN=C(C=C4)C5=C(C=C(C=C5)C(F)(F)F)C(F)(F)F)F	5.20	6.15	7.29	6.89	6.10	[6]
13	mol1	Fc1cc(ccc1F)CCOC=1n2c(nnc2-c2ccc(nc2)C(F)(F)F)C=NC=1	4.48	5.91	5.52	5.11	5.66	[7]
14	mol2	FC(F)Oc1ccc(cc1)-c1n2c(nn1)C=NC=C2CN1Cc2c(C1)cccc2	4.53	4.23	5.14	5.73	5.49	[7]
15	OSM-S-175	FC(F)(F)c1nccc(NC(=O)C=2n3c(nnc3-c3ccc(OC(F)F)cc3)C=NC=2)c1	5.60	5.04	5.64	5.89	4.82	[7]
16	OSM-S-189	Clc1ccccc1CCOC=1n2c(nnc2-c2ccc(cc2)C#N)C=NC=1	4.48	6.03	6.38	6.11	5.02	[7]
17	OSM-S-201	Clc1cccc(NC(=O)C=2n3c(nnc3-c3ccc(OC(F)F)cc3)C=NC=2)c1C	5.12	5.84	5.80	5.26	5.27	[7]
18	OSM-S-202	Clc1cc(NC(=O)C=2n3c(nnc3-c3ccc(OC(F)F)cc3)C=NC=2)ccc1	4.89	5.68	5.75	5.41	5.38	[7]
19	OSM-S-206	FC(F)(F)c1cc(cc(NC(=O)C=2n3c(nnc3-	5.24	5.57	5.73	5.49	4.52	[7]

		c3ccc(OC(F)F)cc3)C=NC=2)c1)C(F)(F)F						
20	OSM-S-218	Fc1cc(ccc1F)[C@@H](OC)COC=1n2c(nnc2-c2ccc(cc2)C#N)C=NC=1	5.20	5.40	6.00	5.73	6.36	7
21	OSM-S-31	O(C(=O)c1cc(n(c1C)-c1ccccc1)C)CC	5.41	5.69	5.40	5.49	4.89	5
22	OSM-S-35	S\1\C(=C/c2cc(n(c2C)-c2ccccc2)C)\C(=O)N/C/1=N/c1ccccc1	4.50	4.43	7.43	5.48	5.65	7
23	OSM-S-353	FC(F)OC(C=C1)=CC=C1C2=NN=C3C=NC=C(OCC(C4=CC=CC=C4)CO)N32	5.12	5.63	6.11	5.92	5.89	7
24	OSM-S-369	FC(F)OC(C=C1)=CC=C1C2=NN=C3C=NC=C(OCCC4=CC=CC=C4)N32	5.43	5.91	5.69	5.40	4.76	7
25	OSM-S-371	FC(F)OC(C=C1)=CC=C1C2=NN=C3C=NC=C(OCCC4(C56)C7C6C8C5C4C87)N32	5.64	5.98	5.95	5.71	5.68	7
26	OSM-S-418	FC(OC1=CC=C(C2=NN=C3C=NC=C(N32)OCC[C]4[CH]BBBBBBBBBB4)C=C1)F	5.92	6.11	7.92	6.39	7.42	7
27	OSM-S-5	Fc1ccc(-n2c(C)c(cc2C)C(OCC(=O)N)=O)cc1	4.50	5.01	5.51	5.89	4.80	7

28	OSM-S-525	FC1=CC(CCOC2=CN=CC3=NN=C(C4=CC=C5C(NC=C5)=C4)N32)=CC=C1F	5.76	5.47	6.43	6.39	5.33	[7]
29	OSM-S-536	FC(F)OC(C=C1)=CC=C1C2=NN=C3C=NC=C(OC[C@H](N)CC4=CC=CC=C4)N32	5.46	6.90	7.15	6.67	5.91	[7]
30	Tegobuvir	C1=CC=C(C(=C1)C2=NC3=CN(C=CC3=N2)CC4=NN=C(C=C4)C5=C(C=C(C=C5)C(F)(F)F)C(F)(F)F)F	6.10	6.86	6.66	6.32	6.14	[6]
	R^2			**0.51**	**0.42**	**0.46**	**0.38**	

References:

1 Higgins, M. A. *et al.* Triazolopyridine ethers as potent, orally active mGlu 2 positive allosteric modulators for treating schizophrenia. *Bioorganic & medicinal chemistry* **25**, 496-513 (2017).

2 Hill, M. D. *et al.* Development of 4-Heteroarylamino-1′-azaspiro [oxazole-5, 3′-bicyclo [2.2. 2] octanes] as α7 Nicotinic Receptor Agonists. *ACS medicinal chemistry letters* **8**, 133-137 (2016).

3 Kim, T. *et al.* Discovery of benzimidazole derivatives as modulators of mitochondrial function: A potential treatment for Alzheimer's disease. *European journal of medicinal chemistry* **125**, 1172-1192 (2017).

4 Shrestha, S. K. *et al.* Bis (N-amidinohydrazones) and N-(amidino)-N′-aryl-bishydrazones: new classes of antibacterial/antifungal agents. *Bioorganic & medicinal chemistry* **25**, 58-66 (2017).

5 Munawar, S. *et al.* Experimentally Validated Pharmacoinformatics Approach to Predict hERG Inhibition Potential of New Chemical Entities. *Frontiers in Pharmacology* **9**, 1035 (2018).

6 Liu, M. *et al.* Design, synthesis, and structure-activity relationships of novel imidazo [4, 5-c] pyridine derivatives as potent non-nucleoside inhibitors of hepatitis C virus NS5B. *Bioorganic & medicinal chemistry* **26**, 2621-2631 (2018).

7 Williamson, A. E. *et al.* Open source drug discovery: highly potent antimalarial compounds derived from the tres cantos arylpyrroles. *ACS central science* **2**, 687-701 (2016).
